# Rapid onset of myocardial calcification following septic shock due to influenza superinfection: a case report

**DOI:** 10.1093/ehjcr/ytae609

**Published:** 2025-02-20

**Authors:** Thibaut Gobé, Adrien Werquin, Nicolas Polge, Bernard Cholley

**Affiliations:** Department of Anesthesiology and Intensive Care Medicine, Hôpital Européen Georges Pompidou, AP-HP, 20 Rue Leblanc 75015, Paris, France; Department of Anesthesiology and Intensive Care Medicine, Hôpital Européen Georges Pompidou, AP-HP, 20 Rue Leblanc 75015, Paris, France; Department of Anesthesiology and Intensive Care Medicine, Hôpital Européen Georges Pompidou, AP-HP, 20 Rue Leblanc 75015, Paris, France; Department of Anesthesiology and Intensive Care Medicine, Hôpital Européen Georges Pompidou, AP-HP, 20 Rue Leblanc 75015, Paris, France; Université Paris Cité, Inserm UMR_S 1140, F-75006 Paris, France

**Keywords:** ARDS, VV-ECMO, Subepicardial calcification, Myocardial calcification, Septic shock, Global longitudinal strain, Case report

## Abstract

**Background:**

Myocardial calcification is an unusual complication of septic cardiomyopathy. Dystrophic calcification may occur after damage to myocardial tissue, but its occurrence is rare.

**Case summary:**

A 42-year-old pregnant woman with no past medical history was admitted in intensive care for respiratory distress caused by bacterial superinfection of influenza. Subepicardial calcification of the left ventricle appeared on a computed tomography at Day 8. Calcification worsened quickly, but we observed a paradoxical improvement in left ventricle function, as assessed by left ventricular ejection fraction and global longitudinal strain using echocardiography with speckle tracking.

**Discussion:**

The pathophysiology of myocardial calcification is not fully understood, but prolonged haemodynamic failure, profound acidosis, high vasopressor doses, and acute respiratory distress syndrome were previously associated with septic-related myocardial calcification.

**Learning points**

- Myocardial calcification may appear during the first week after the onset of severe septic shock.
- Left ventricle global longitudinal strain may be preserved despite extensive myocardial calcification. Sinus node dysfunction may occur during the late time course of this unusual complication of septic shock.

## Introduction

Septic cardiomyopathy is reported in 30–60% of patients with septic shock,^1^ but onset of diffuse myocardial calcification is an unusual complication in this setting with <20 case reports in the literature since the year 2000. Myocardial calcification may occur over the first few days and up to 1 year after the onset of septic shock.^2,3^

This myocardial calcification is called either ‘dystrophic’ when occurring in damaged tissue or ‘metastatic’ when it is secondary to abnormal calcium homoeostasis, such as in patients with chronic renal failure on haemodialysis, hyperparathyroidism, or oxaluria.^4^ Myocardial calcification has previously been associated with altered contractility and/or relaxation, leading to systolic and/or diastolic dysfunction.^4^

In this report, we describe the case of a pregnant woman with a rapid onset of dystrophic myocardial calcification following septic shock due to influenza superinfection.

## Summary figure

**Figure:**
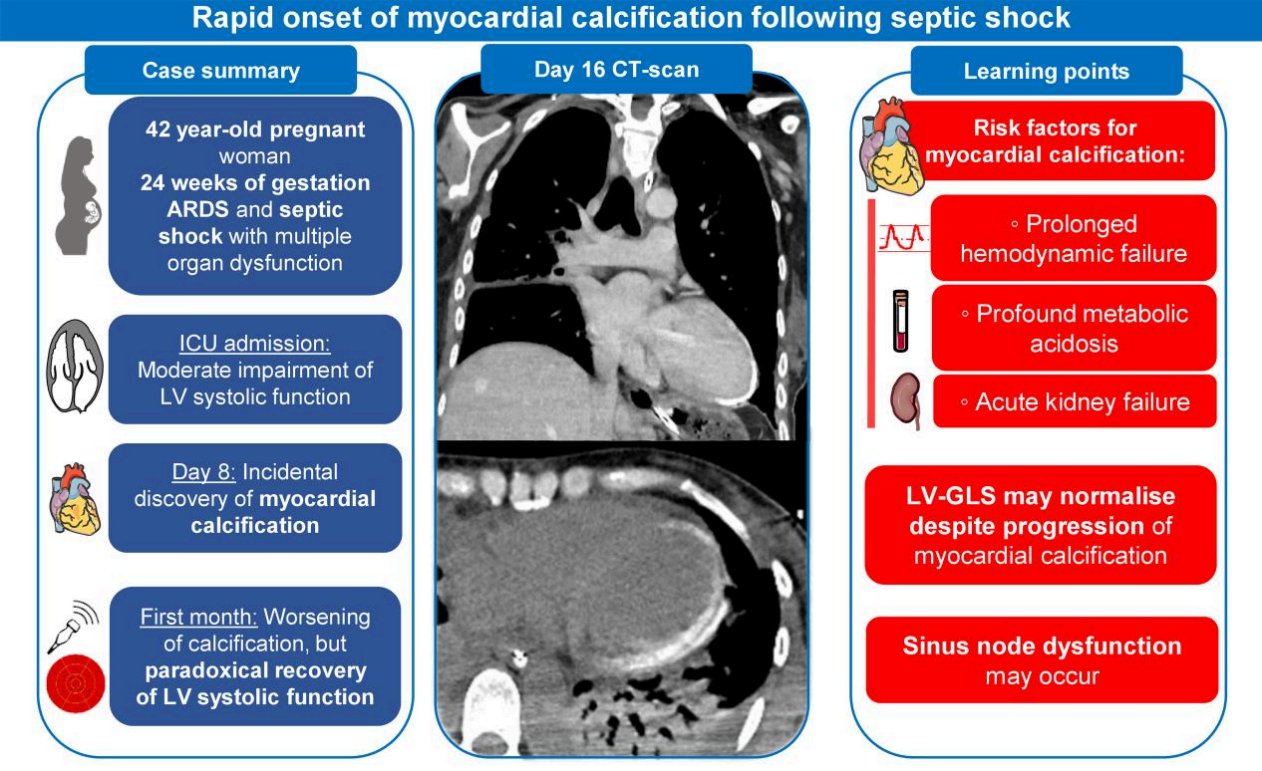


## Case presentation

A 42-year-old pregnant woman (G2P1) at 24 weeks of gestation with no past medical history, except a laparoscopic exploration for endometriosis, was admitted for acute respiratory distress. She presented with cough, fever, and dyspnoea 1 week before admission. She had received symptomatic treatment with acetaminophen. Vital parameters at admission were as follows: heart rate 125 b.p.m., arterial pressure 80/45 mmHg, SpO2 80% on room air, respiratory rate 40/min, temperature 38°C. electrocardiogram within 24 h of admission was normal (see Supplementary material online, *Figure S1*). Heart auscultation revealed regular tachycardia but no abnormal heart sound. Diffuse crackles were heard throughout both lung fields.

**Figure 1:**
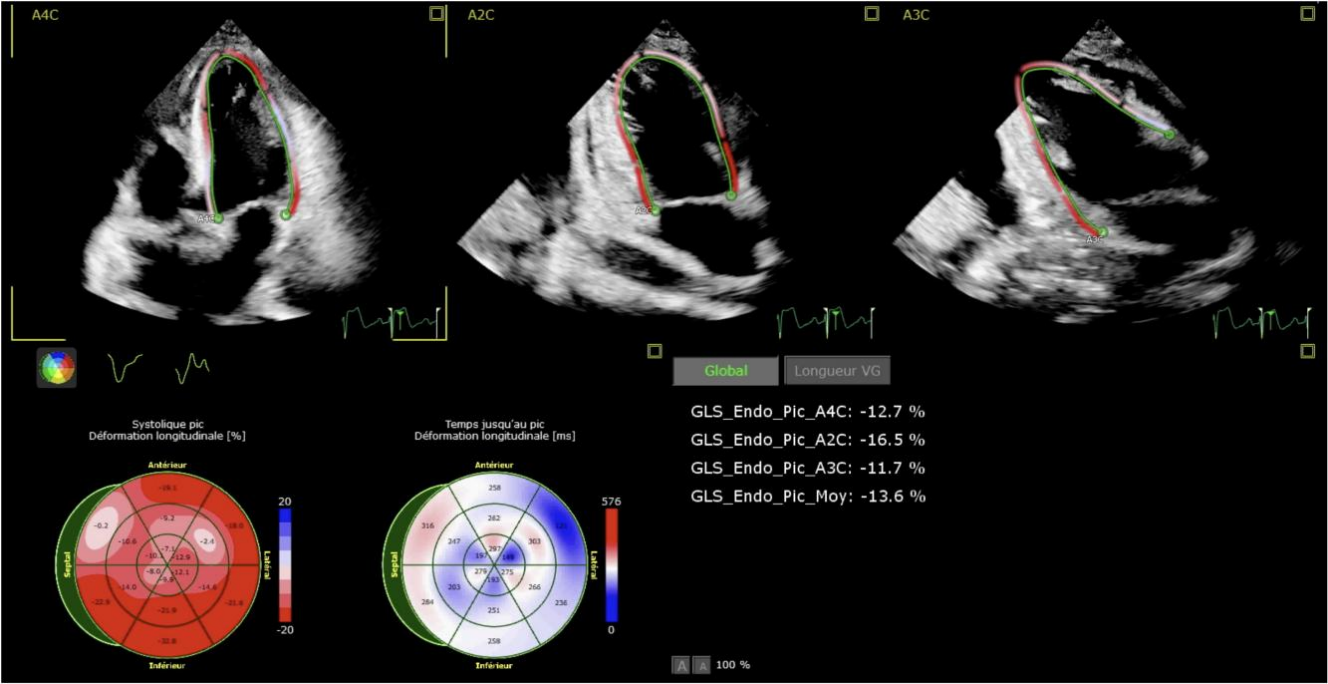
Global longitudinal strain for left ventricular function at Day 1.

Transthoracic echocardiography revealed mild left ventricular (LV) dysfunction [LV ejection fraction (LVEF): 45%, LV outflow tract velocity-time integral (LVOT VTI): 17 cm; LV global longitudinal strain (LV-GLS): −13.6%] (*Figure 1*; Supplementary material online, *Video S1*), abnormal relaxation according to the mitral Doppler pattern (E: 80 cm/s; A: 118 cm/s; E/A: 0.7; lateral e′: 7.1 cm/s; E/e′: 11.3), and a small non-compressive pericardial effusion.

The chest X-ray showed bilateral alveolar infiltrates and the thoracic computer tomography confirmed consolidation of both lower lobes. *Streptococcus pneumoniae* antigen was present in urine, and respiratory polymerase chain reaction was positive for influenza virus A. In addition, a blood culture grew positive to *Pseudomonas aeruginosa*. The patient was diagnosed as having a bacterial superinfection of influenza. An intravenous anti-infectious treatment was initiated with a combination of piperacillin–tazobactam (4 g bolus followed by a continuous infusion of 16 g/24 h for 7 days), spiramycin (3 million units 3 times a day for 3 days, i.e. until *Legionella pneumophila* was ruled out by negative urine antigen), amikacin (one single dose of 2 g), and oseltamivir (75 mg 2 times a day for 5 days).

Despite high-flow nasal oxygen and intermittent non-invasive positive pressure ventilation, hypoxaemia persisted and required orotracheal intubation with deep sedation, muscle relaxation, and protective mechanical ventilation. Gas exchange continued to deteriorate. A support using veno-venous extra-corporeal membrane oxygenation (VV-ECMO) was introduced, and three sessions of prone positioning were required.

Septic shock was responsible for intrauterine foetal death and for multiple organ failure with a score of 19 on the sepsis-related organ failure assessment scale [range (0–24), predicted mortality > 90%] at intensive care unit admission. Acute kidney failure was managed using continuous veno-venous haemodialysis (CVVHD) with regional citrate anticoagulation. Additionally, the patient presented with acute liver failure {bilirubin: 249 µmol/L [normal value (*N*) < 21 µmol/L]; Prothrombin time: 22% [*N* > 65%]}, severe vasoplegia requiring high dose of vasopressors (norepinephrine 4 μg/kg/min and vasopressin 0.03 UI/min), and mild LV dysfunction (LVEF: 45%) attributed to flu myocarditis [high-sensitivity troponin I plasma level: 43 000 ng/L (*N* < 11.6 ng/L)].

**Figure 2:**
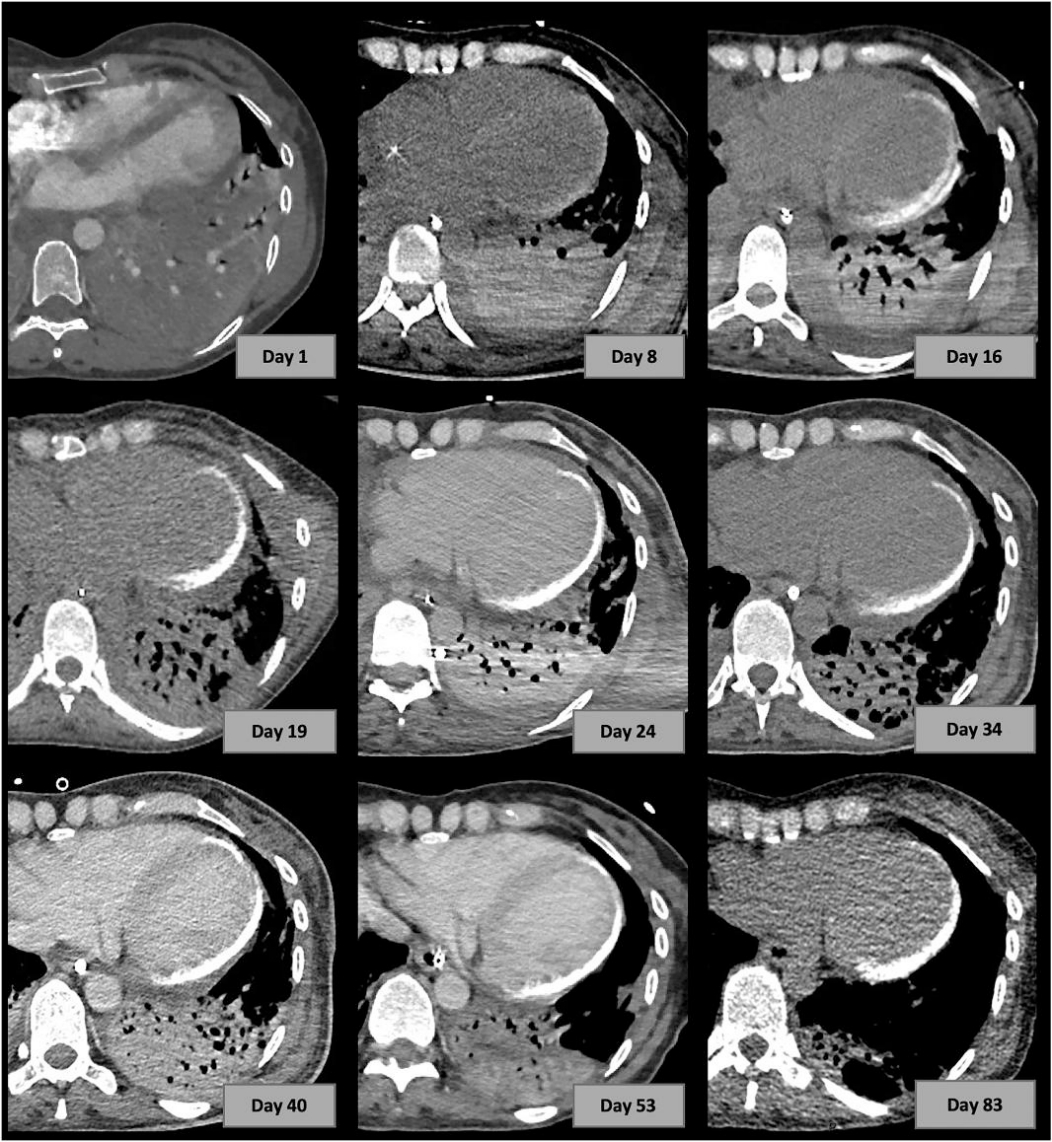
Evolution in myocardial calcification on the serial chest computed tomography scans performed over the time course of the acute respiratory distress syndrome.

On Day 16, a lung computed tomography (CT) scan revealed extensive subepicardial calcification of the free wall of the LV. Retrospectively, this calcification was detectable, although less pronounced, on the CT scan at Day 8. Repeated CT scans were performed regularly to evaluate the lung parenchyma and the progression of myocardial calcification (*Figure 2*). Furthermore, other calcium deposits appeared in the left masseter muscle and the right shoulder capsule.

Hyperparathyroidism was ruled out based on low plasma levels for parathormone [8 pg/mL (*N* = 11–57 pg/mL)] and vitamin D [12.8 pg/mL (*N* = 19.9–79.3 pg/mL)] and normal values for calcium [2.23 mmol/L (*N* = 2.20–2.65 mmol/L)].

On Days 29 and 31, two episodes of sinus node dysfunction occurred (see Supplementary material online, *Figure S2*), requiring cardiopulmonary resuscitation. A leadless pacemaker was implanted.

**Figure 3:**
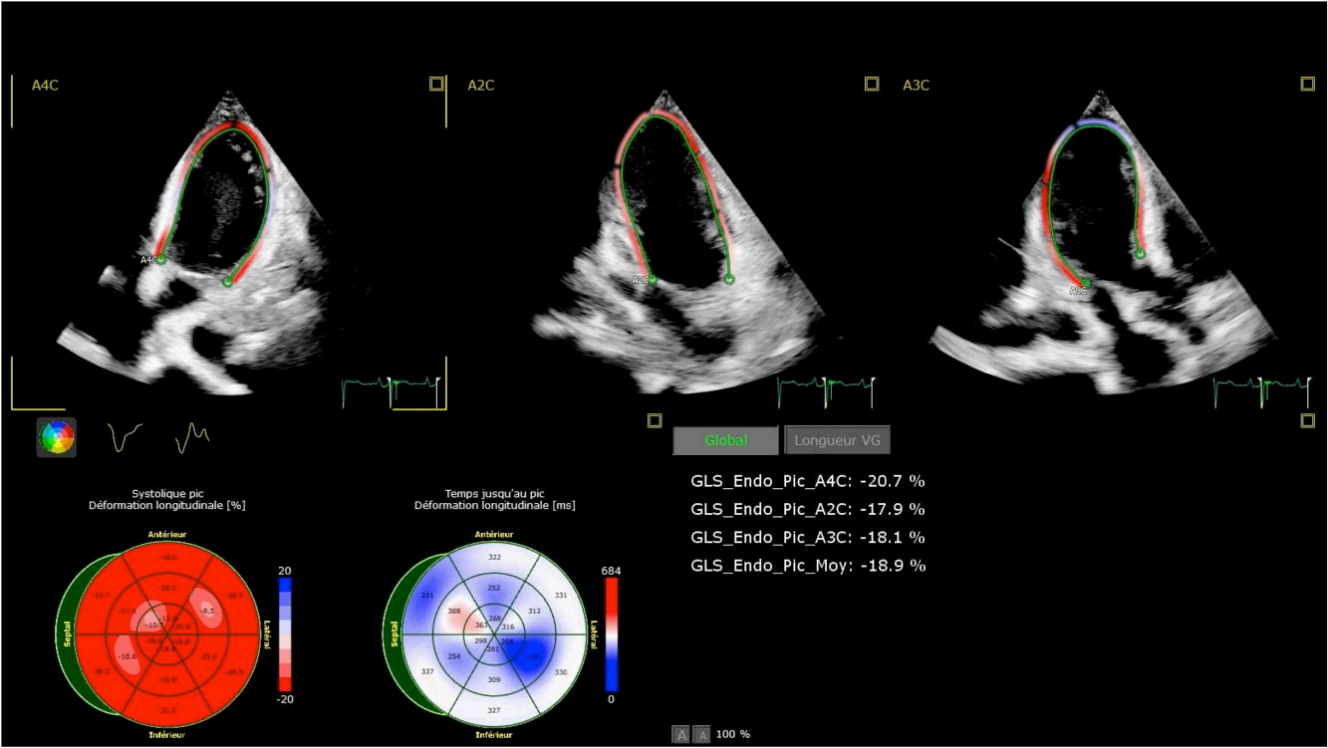
Global longitudinal strain for left ventricular function at Day 8.

From admission onwards, daily echocardiographic evaluation (including LV-GLS) was performed to monitor LV systolic function. Complete recovery of LV function was observed at Day 8 [LVEF: 55%; LVOT VTI: 22 cm; LV-GLS: −18.9% (*Figure 3*; Supplementary material online, *Video S2*); E: 52.3 cm/s; A: 59.6 cm/s; E/A: 0.9; lateral e′: 10.1 cm/s; E/e′: 5.2]. An apical four-chamber view without speckle-tracking analysis showing LV lateral wall is provided as Supplementary material online, *Video S3*.

Improvement of all organ failure allowed the patient to be discharged to a rehabilitation unit on Day 44, despite a worsening in myocardial calcification.

Two months after discharge, a cardiac CT angiography found a stabilization of myocardial calcification and no coronary lesion.

At 1 year after discharge, electrocardiogram showed a sinus rhythm (see Supplementary material online, *Figure S3*) and the stimulation rate from the leadless pacemaker at several follow-up visits was <1%. A follow-up transthoracic echocardiographic examination was completely normal and ruled out signs of constriction (LVEF: 60%; LV-GLS: −19%; E: 92 cm/s; A: 35 cm/s; E/A: 2.6; medial e′: 10.7 cm/s; lateral e′: 11.4 cm/s; E/e′: 8) absence of pericardial effusion. A cardiac MRI was also performed showing a late subepicardial enhancement of the lateral wall of the LV, consistent with a recent myocarditis, but chamber volumes and ventricular function were normal (see Supplementary material online, *Figure S4*).

## Discussion

In this patient with severe septic shock, we observed an early onset of extensive myocardial calcification, without any detectable metabolic disorder in the context of regional citrate anticoagulation of the CVVHD. The high troponin plasma levels at admission were consistent with acute myocarditis. Therefore, we qualified this calcification as ‘dystrophic’.

Very few cases of myocardial calcification have been reported in the context of VV-ECMO or VA-ECMO for myocarditis.^2,3,5,6^ The pathophysiology is not fully understood, but prolonged haemodynamic failure, profound acidosis, high vasopressor doses, and acute respiratory distress syndrome (ARDS) seemed associated with sepsis-related calcification.^2^ Although initially barely detectable, myocardial calcification became visible as early as Day 8, which is quite unusual.^7^

Myocardial calcification has previously been associated with altered contractility and/or relaxation, leading to systolic and/or diastolic dysfunction.^4^ Interestingly, in the present case, longitudinal strain was preserved and our patient did not develop any diastolic or constrictive dysfunction. This lack of significant change in LV longitudinal shortening has only been reported once previously.^8^ Although LV-GLS was abnormal (−13.6%) at admission in our patient, daily measurements showed a full recovery of this parameter within 10 days, despite simultaneous progression in the epicardial calcification. Left ventricle global longitudinal strain values obtained in mechanically ventilated patients should be considered with caution due to the difficulty in obtaining perfect endocardial detection on the three required views. However, in the present case, LV-GLS variations reflected simultaneous changes in LVEF, supporting the paradoxical improvement in LV function. The lack of correlation between LV-GLS and the progression of myocardial calcification can probably be explained, in part, by the fact that this index is derived from shortening within the endocardium whereas calcification occurred in the subepicardium. However, the dissociation between the massive calcification of the epicardium with the simultaneous improvement in endocardial longitudinal shortening was striking and has not yet been described.

The calcification can also affect the conductive pathway, and some authors have reported sudden death associated with calcium deposits in conductive tissue of patients with chronic kidney failure.^9,10^ Our patient had recovered from acute kidney injury after 27 days of renal replacement therapy when she presented with two episodes of sinus node dysfunction responsible for circulatory arrest. Our initial hypothesis was that calcium deposits in the conductive tissue were likely responsible for this life-threatening conduction disorder, since there was no trigger for vasovagal episode. We considered that an interim method such as temporary pacing would not have been sufficient given the irreversibility of myocardial calcification, and we decided on emergency permanent pacemaker implantation. At that time, our patient was too unstable to have an electrophysiologic study. Retrospectively, since the patient recovered a spontaneous sinus rhythm, this hypothesis was probably erroneous and a vagal mechanism might have been responsible. The concomitant calcification of the right shoulder and the left masseter muscle is also very unusual in this context.

Even though calcium plasma levels were always normal in our patient, the regional citrate anticoagulation of CVVHD (requiring continuous calcium infusion) may have played a role in the evolution of myocardial calcification. We decided to switch anticoagulation from regional citrate to general unfractionated heparin, hypothesizing potential harmfulness of this technique in this specific situation.

## Conclusion

Subepicardial calcification is a rare complication after severe septic shock. This case stands out by the early onset and remarkable rapidity of evolution, by the striking contrast between intensity of calcification and preservation of LV systolic and diastolic function.

The role of calcium citrate regional anticoagulation in the development of this complication is hypothetical and requires more observations to suggest a possible participation.

## Supplementary Material

ytae609_Supplementary_Data

## Lead author biography

Thibaut Gobé, MD, graduated from the Faculty of Medicine of the University of Paris in 2023, is currently specializing in cardiovascular critical care.
