# Supplementary material for: Rapid onset of myocardial calcification following septic shock due to influenza superinfection: a case report
Source: Eur Heart J Case Rep. 2025 Feb 20;9(2):ytae609. doi: 10.1093/ehjcr/ytae609 (PMC11839270; doi:10.1093/ehjcr/ytae609)
Supplement: ytae609_Supplementary_Data [file ytae609_supplementary_data.zip › Supplemental files - review 3.docx]

**Supplemental files legends**

**Supplemental Figure 1**: ECG at day 1.

**Supplemental Figure 2**: Trace recording showing 3 ECG leads and invasive arterial blood pressure waveform during sinus node dysfunction on day 29.

**Supplemental Figure 3**: ECG at day 480.

**Supplemental Figure 4:** cardiac MRI images (T2 sequence) at 1 year: 4-chamber and 2-chamber views.

**Supplemental Video 1:** LV-GLS at day 1.

**Supplemental Video 2**: LV-GLS at day 8.

**Supplemental Video 3:** Apical 4-chamber view obtained at day 27, when myocardial calcification of the lateral wall was obvious on CT images. Lateral wall epicardial calcification was clearly not detectable using ultrasound.


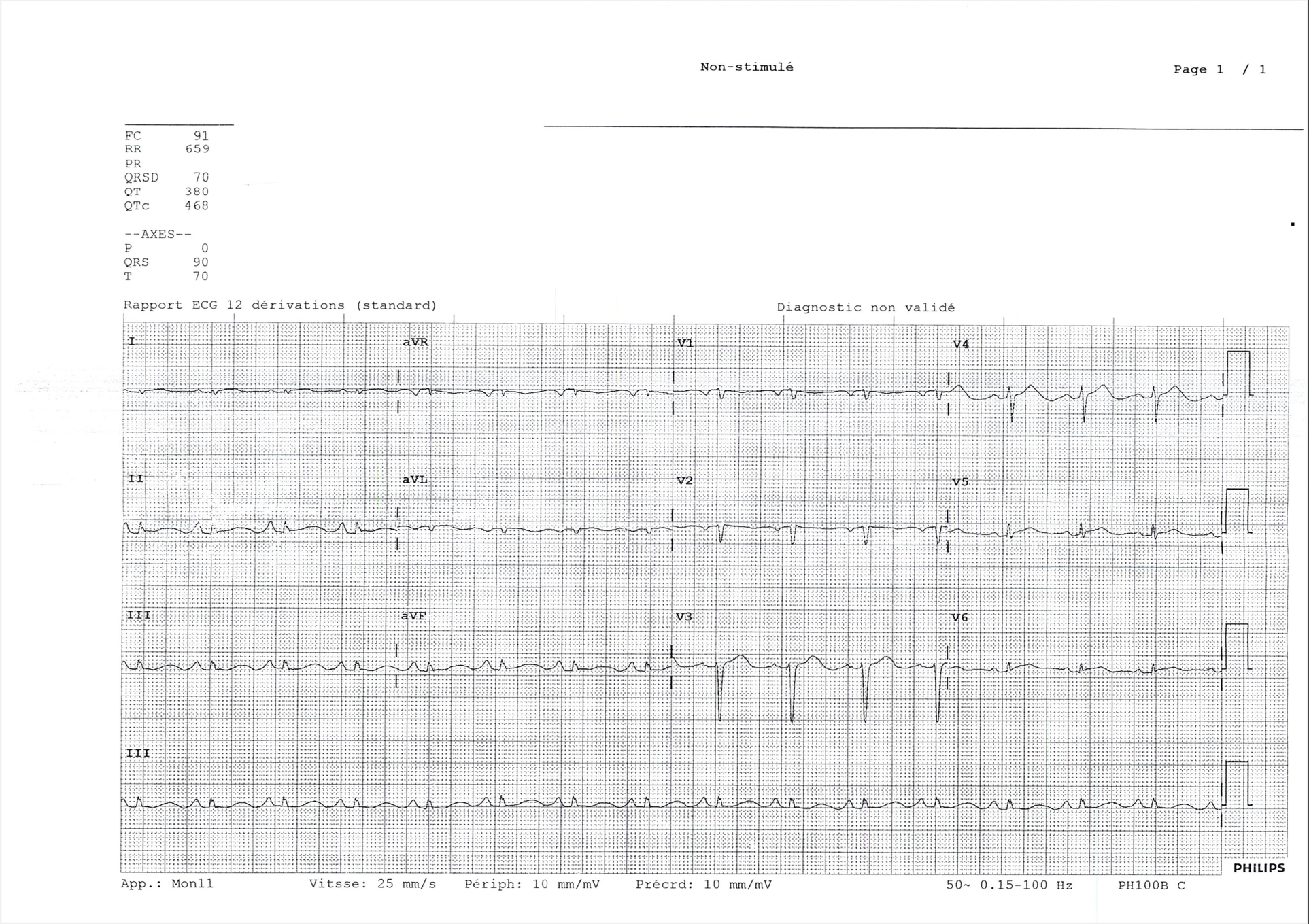


**Supplemental Figure 1**: ECG at day 1.


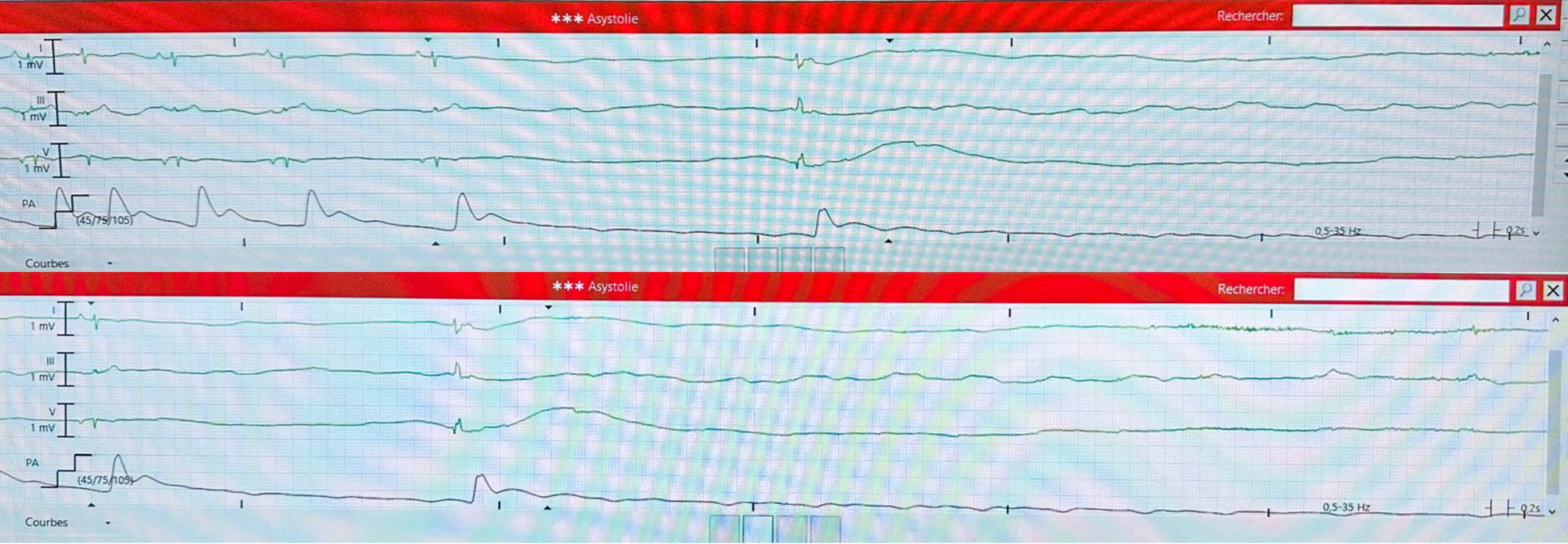


**Supplemental Figure 2**: Trace recording showing 3 ECG leads and invasive arterial blood pressure waveform during sinus node dysfunction on day 29.


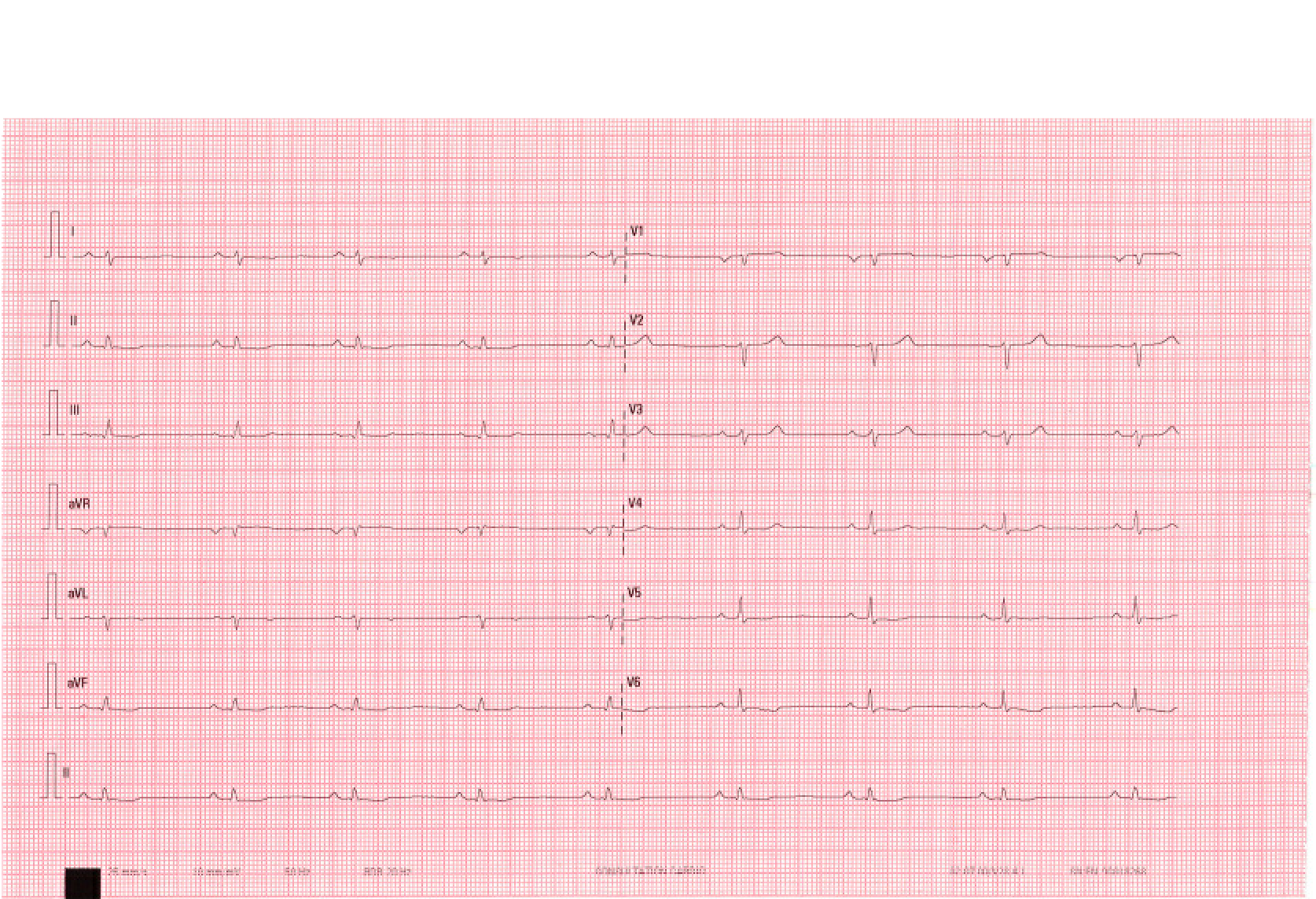


**Supplemental Figure 3**: ECG at day 480.


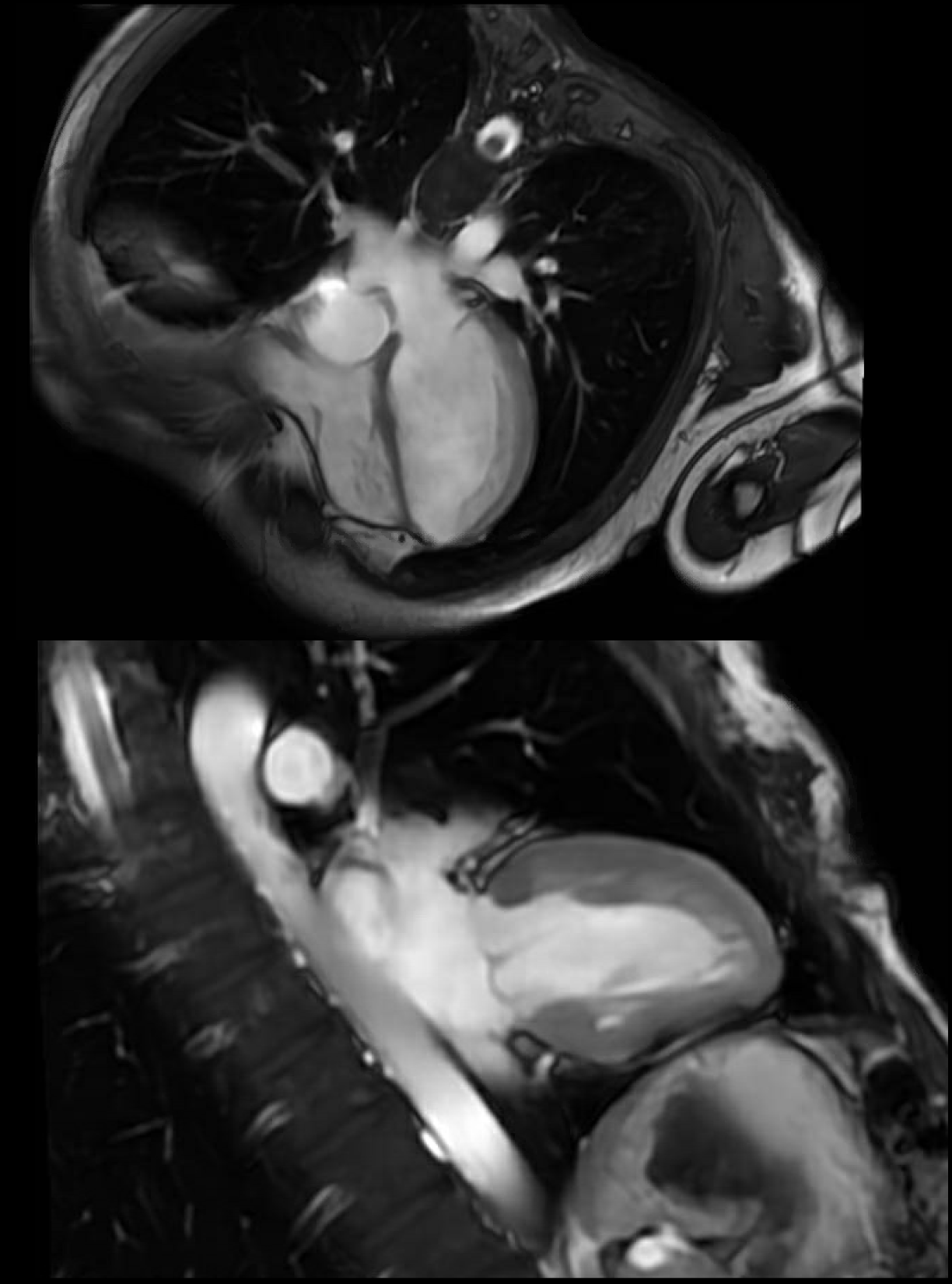


**Supplemental Figure 4:** cardiac MRI images (T2 sequence) at 1 year : 4-chamber and 2-chamber views.

**
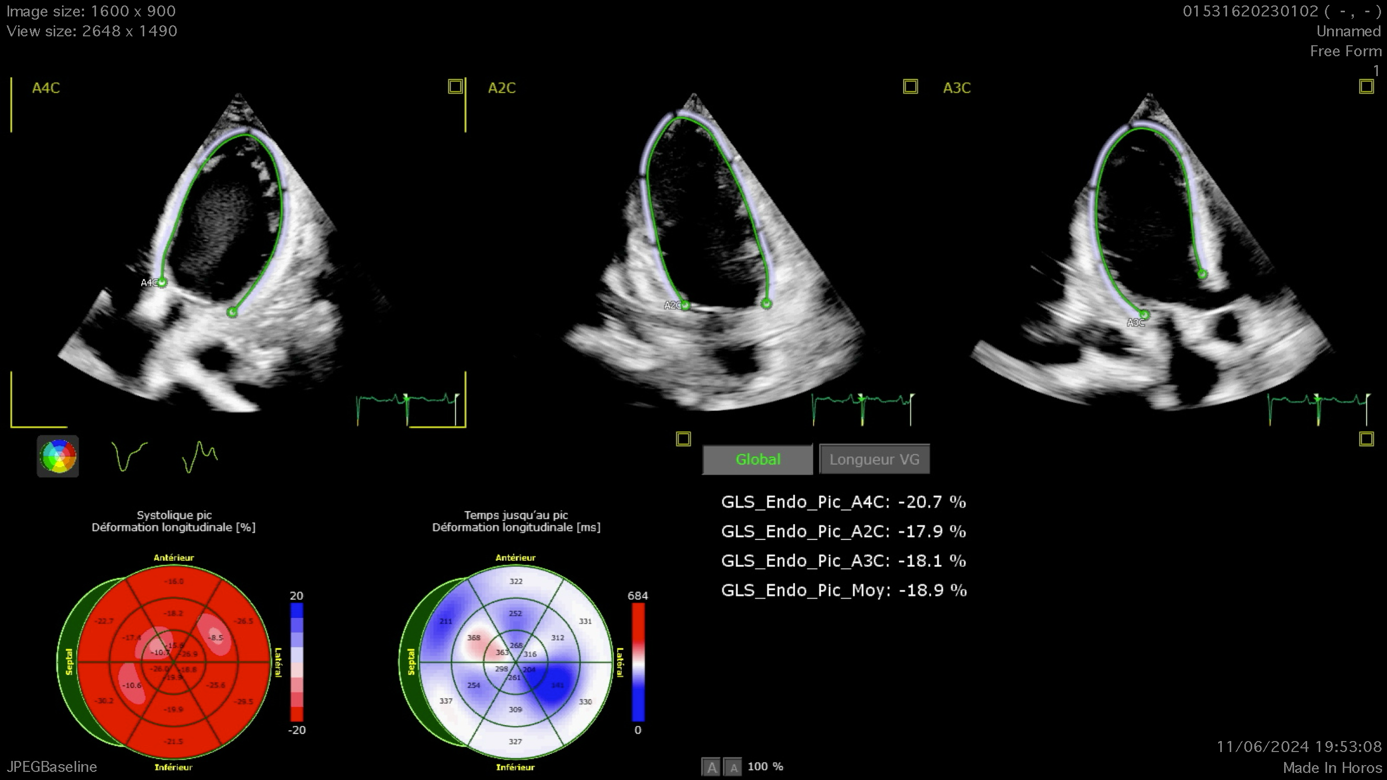

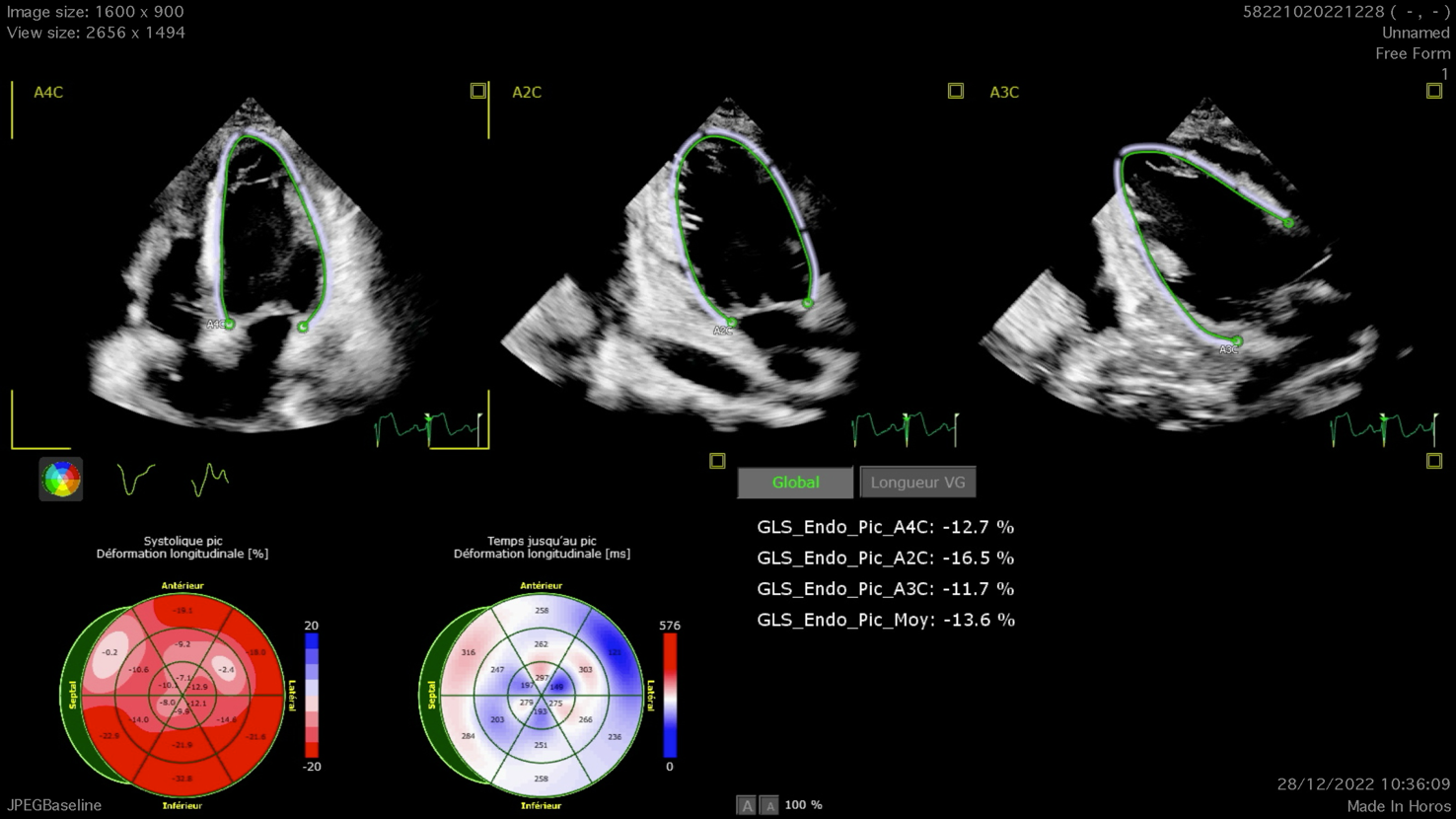
**

**Supplemental Video 1:** LV-GLS at day 1.

**Supplemental Video 2**: LV-GLS at day 8.

**
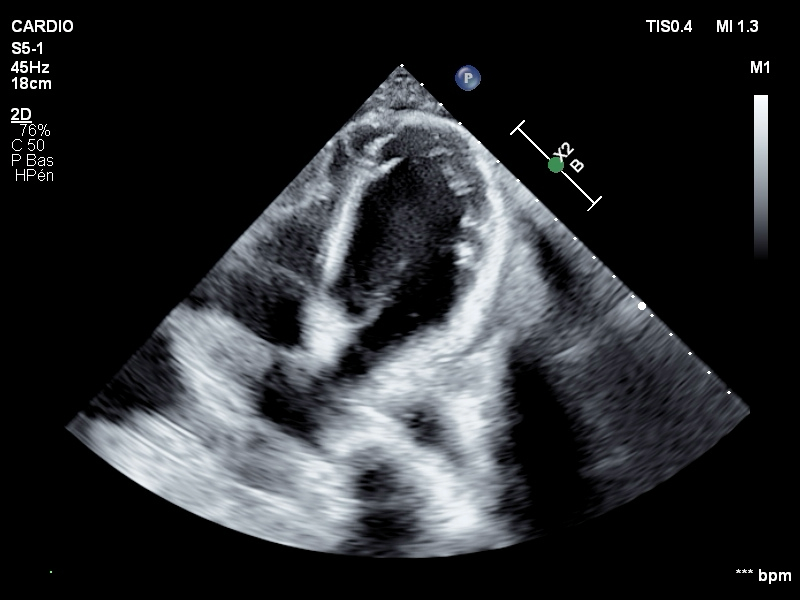
**

**Supplemental Video 3:** Apical 4-chamber view obtained at day 27, when myocardial calcification of the lateral wall was obvious on CT images. Lateral wall epicardial calcification was clearly not detectable using ultrasound.
