# Supplementary figures and images for: Rapid onset of myocardial calcification following septic shock due to influenza superinfection: a case report
Source: Eur Heart J Case Rep. 2025 Feb 20;9(2):ytae609. doi: 10.1093/ehjcr/ytae609 (PMC11839270; doi:10.1093/ehjcr/ytae609)

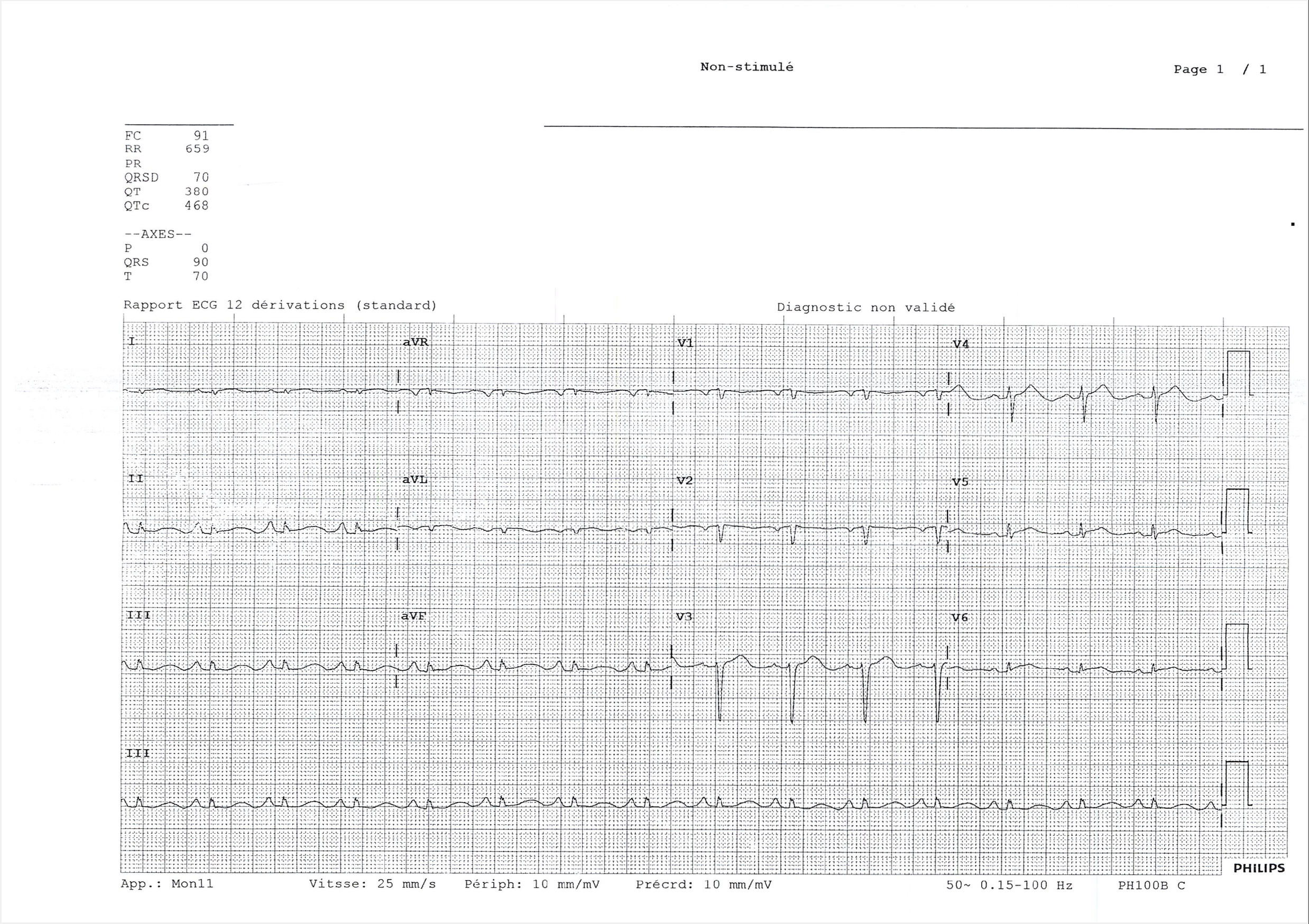

Supplement: ytae609_Supplementary_Data [file ytae609_supplementary_data.zip › Supplemental figure 1.tiff]

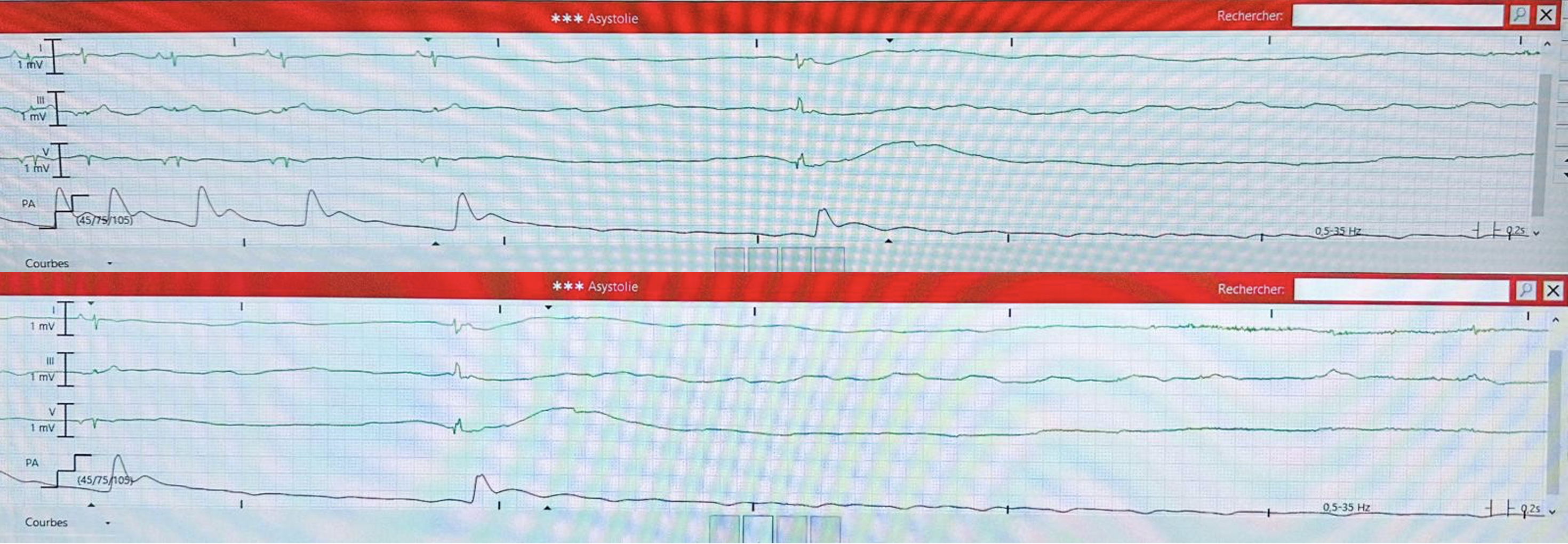

Supplement: ytae609_Supplementary_Data [file ytae609_supplementary_data.zip › Supplemental figure 2.tiff]

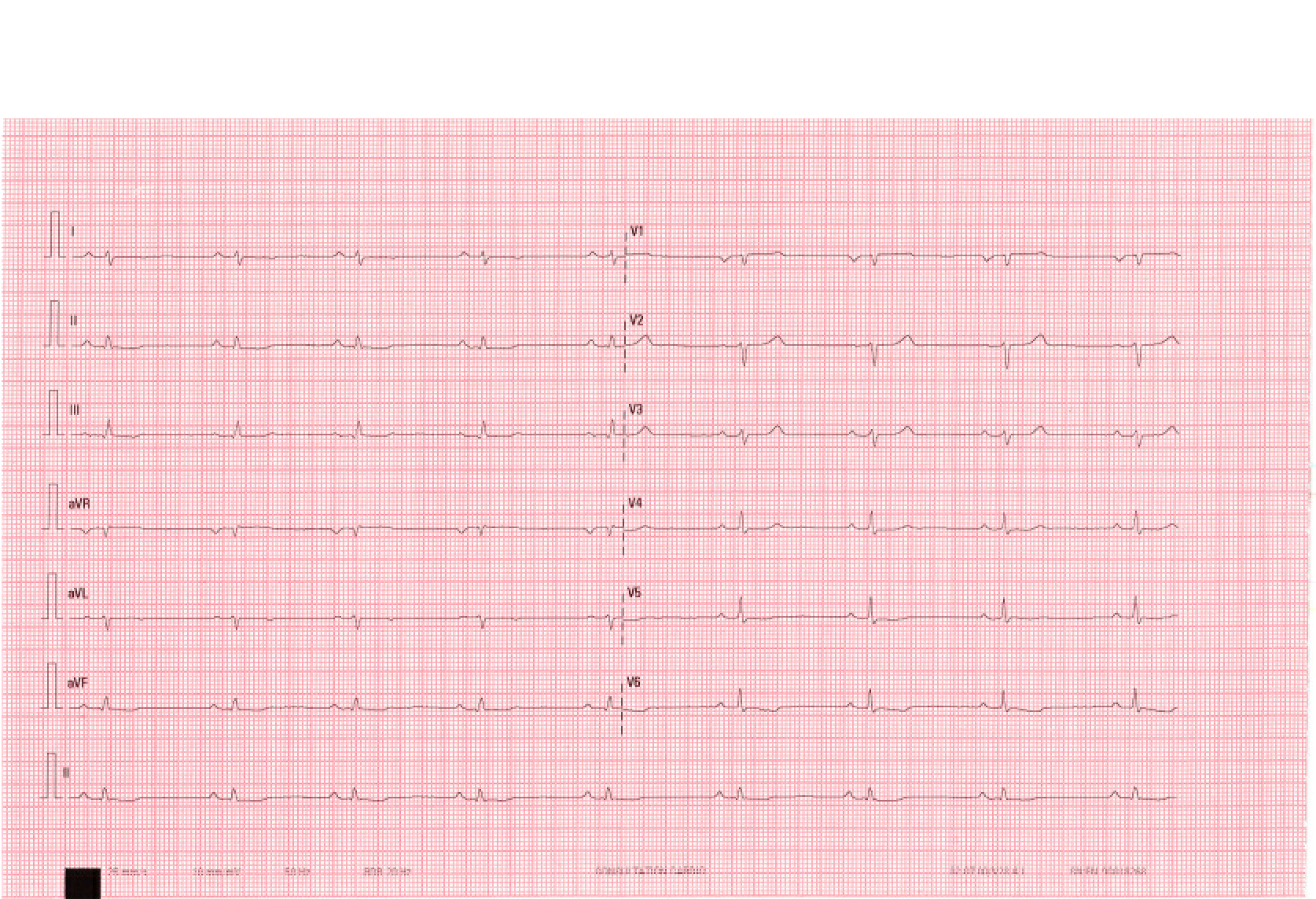

Supplement: ytae609_Supplementary_Data [file ytae609_supplementary_data.zip › Supplemental figure 3.tiff]

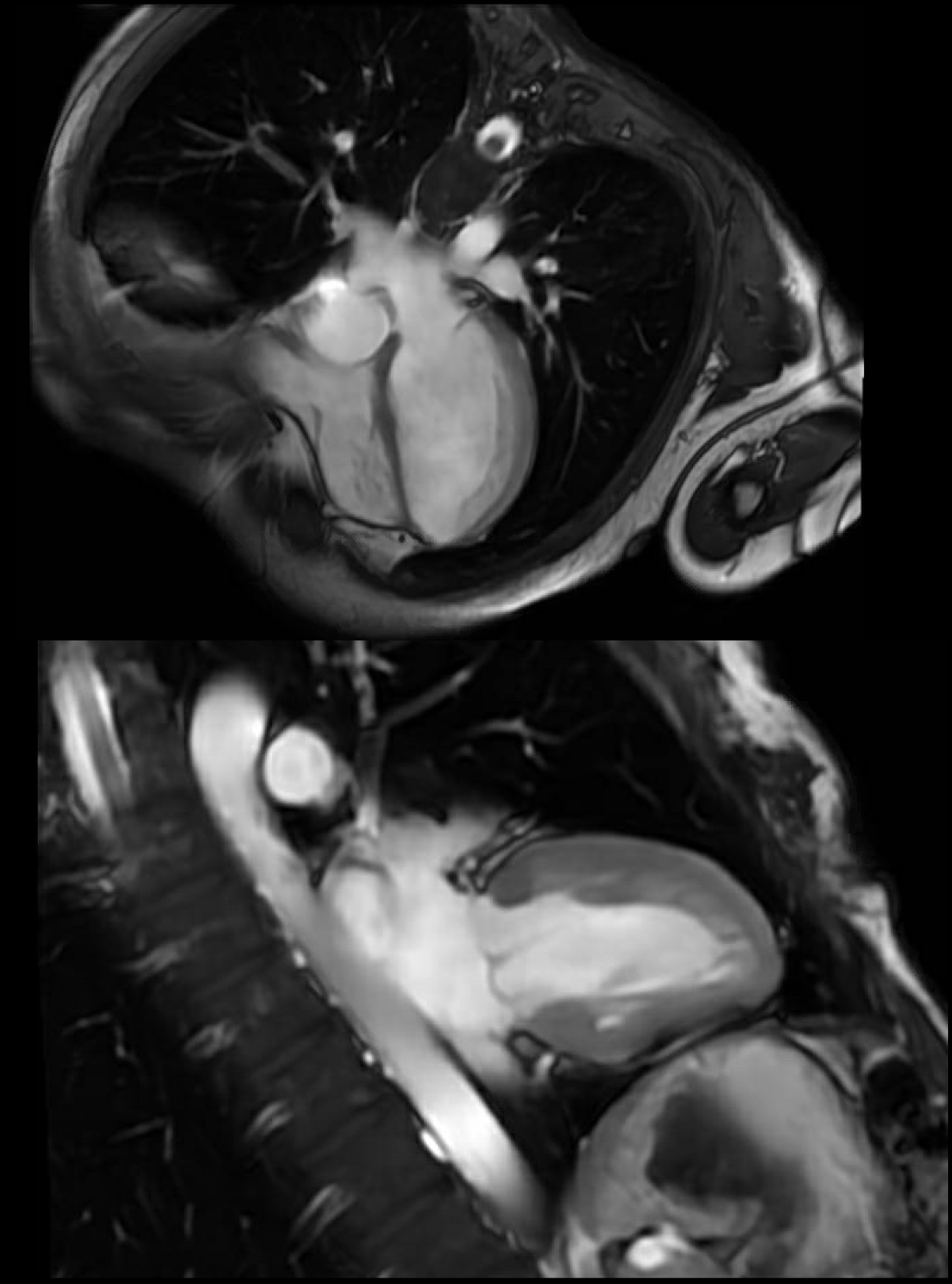

Supplement: ytae609_Supplementary_Data [file ytae609_supplementary_data.zip › Supplemental figure 4.tiff]
